# Mechanisms of bone pain: Progress in research from bench to bedside

**DOI:** 10.1038/s41413-022-00217-w

**Published:** 2022-06-06

**Authors:** Gehua Zhen, Yuhan Fu, Chi Zhang, Neil C. Ford, Xiaojun Wu, Qichao Wu, Dong Yan, Xueming Chen, Xu Cao, Yun Guan

**Affiliations:** 1 Department of Orthopedics, Johns Hopkins University, School of Medicine, Baltimore, MD 21205 USA; 2 Department of Anesthesiology and Critical Care Medicine, Johns Hopkins University, School of Medicine, Baltimore, MD 21205 USA; 3 Division of Pathology, Sibley Memorial Hospital Washington, Washington, DC 20016 USA; 4 Department of Oncology, Beijing Luhe Hospital, Capital Medical University, Beijing, 100149 China; 5 Department of Orthopedics, Beijing Luhe Hospital, Capital Medical University, Beijing, 100149 China; 6 Department of Neurological Surgery, Johns Hopkins University, School of Medicine, Baltimore, MD 21205 USA

**Keywords:** Pathogenesis, Diseases

## Abstract

The field of research on pain originating from various bone diseases is expanding rapidly, with new mechanisms and targets asserting both peripheral and central sites of action. The scope of research is broadening from bone biology to neuroscience, neuroendocrinology, and immunology. In particular, the roles of primary sensory neurons and non-neuronal cells in the peripheral tissues as important targets for bone pain treatment are under extensive investigation in both pre-clinical and clinical settings. An understanding of the peripheral mechanisms underlying pain conditions associated with various bone diseases will aid in the appropriate application and development of optimal strategies for not only managing bone pain symptoms but also improving bone repairing and remodeling, which potentially cures the underlying etiology for long-term functional recovery. In this review, we focus on advances in important preclinical studies of significant bone pain conditions in the past 5 years that indicated new peripheral neuronal and non-neuronal mechanisms, novel targets for potential clinical interventions, and future directions of research.

## Introduction

Bone diseases and injuries are often associated with devastating pain which causes significant suffering and impairs quality of life. Pain may also exacerbate other comorbidities of bone diseases, delay natural wound and bone healing, and disrupt normal bone remodeling and functional recovery^1–5^. The cost of treatment and lost productivity due to bone pain generate a heavy economic burden for patients and society. Unfortunately, current pharmacologic therapies for bone pain have often been associated with suboptimal efficacy and short-lived therapeutic effects. Long-term treatment, such as opioids, often leads to prominent dose-limiting side effects such as addiction and abuse^6–8^. In addition, opioids may exert detrimental effects on bone remodeling and repairing^5,9–11^. Accordingly, it is important to improve our understanding of the respective underlying mechanisms of different bone pain conditions, as the first step towards developing new, non-opioid bone pain therapies in the future.

The current review highlights recent progress in pre-clinical research on significant bone pain conditions from 2015 to 2021. We summarized important findings of the biological basis by which bone pathological changes may produce pain, especially those indicating roles of bone remodeling, peripheral neuronal, and non-neuronal mechanisms. We also reviewed popular animal models of bone pain currently being used and discussed potential challenges and limitations in the study of bone pain using animal models. In the future perspective, we discussed potential novel targets for treatment and directions for future investigation. Because of the large number of bone pain conditions and complicated etiology in each, our review emphasized bone pain associated with skeletal diseases including osteoarthritis, low back pain, autoimmune diseases, and bone metastasis which remain significant clinical problems and difficult to treat. We also briefly reviewed two other bone pain conditions resulted from trauma and aging.

## Bone remodeling and the interactions between bone and the nervous system

### Bone remodeling

Bone remodeling is an important mechanism to maintain healthy bone density and homeostasis^12–14^. Bone is a highly vascularized and innervated connective tissue that constantly undergoes a finely balanced and coupled remodeling process. This process is orchestrated by the activities of osteoclasts, osteoblasts, and osteocytes. Osteoclasts are specialized cells that can resorb the host tissue. Osteoblasts originating from mesenchymal stem cells (MSCs) are the primary bone-forming cells that reconstruct the resorption pits generated by osteoclasts. Osteocytes are the terminally differentiated osteoblasts and the dominant cell type embedded in the bone matrix. In normal conditions, bone remodeling is perfectly balanced in a timely and spacial manner owing to the precise interactions of hormones, paracrine growth factors, and the cooperation of different cell populations.

Bone is highly responsive to mechanical stress and up to 20% of the skeleton undergoes remodeling at any time to repair damage from mechanical stress, and to maintain the integrity of the skeleton^15^. Osteocytes can sense and transmit information of mechanical signals, the presence of microfractures, and extracellular mineral concentrations to the bone surface through their long cytoplasmic processes and the extensive lacuna-canalicular network. After receiving the information from osteocytes, osteoclasts start to resorb bone at the designated site by secreting protons and hydrolytic enzymes. During bone resorption, various growth factors and cytokines such as transforming growth factor-beta (TGF-β) and insulin-like growth factor-1 (IGF-1) are released from the bone matrix. These factors play essential roles in the process of bone remodeling by contributing to MSCs recruitment, osteoblast commitment, and differentiation^16,17^. Subsequently, osteoblasts begin to build new bone at the resorption site by producing osteoid and regulating the deposition of minerals. Osteoblasts regulate the differentiation and maturation of osteoclasts by secreting colony-stimulating factor (M-CSF), receptor activator of nuclear factor-κB ligand (RANKL), and osteoprotegerin (OPG)^18–20^. Mature osteoclasts in turn stimulate osteoblastic bone formation and mineralization by secreting vesicular RANK^21^. A recent study showed that the prostaglandin-E2 (PGE2) secreted by osteoblasts can bind to the EP4 receptor in the sensory nerve, and trigger neuronal activities that regulate bone formation^22^. The structural integrity and mechanical homeostasis of bone substantially rely on this coupled bone remodeling. In contrast, dysregulation of bone remodeling is presented in most skeletal diseases, i.e., osteoporosis, osteoarthritis, degenerative disc diseases, and cancer metastasis.

### The role of the nervous system in regulating bone remodeling

**Fig. 1:**
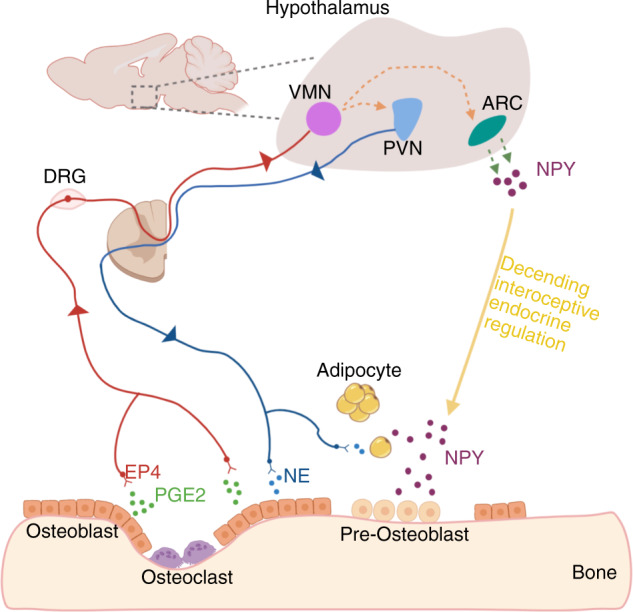
The interoception of bone and EP4-hypothalamus-sympathetic axis in regulating bone formation. The secretion of prostaglandin-E2 (PGE2) by osteoblasts is increased when osteoclast bone resorption occurs and bone density decreases. PGE2 binds to the prostaglandin E receptor 4 (EP4) on sensory nerve fibers, and the signals are relayed to the hypothalamus by afferent nerves. As a result, sympathetic tone is tuned down and the expression of hypothalamic neuropeptide Y (NPY) is downregulated and induces lipolysis of adipose tissue for osteoblastic bone formation.

Bone is richly innervated like most other organs in the body. Sensory and autonomic nerve fibers have been identified in both bone marrow and periosteum, and are particularly concentrated in areas with high osteogenic activity. The vital roles of the nervous system in regulating bone remodeling have entered the scene in recent decades, beginning with the observation of high bone mass in leptin-deficient mice^23^. Yet, mice with conditional knockout of leptin receptors in osteoblasts did not exhibit any abnormal bone phenotype. Thus, it was suggested that leptin may regulate bone mass by acting on ventromedial hypothalamic neurons^24^. Specifically, upon receiving stress signals such as exercise and temperature change, the hypothalamic neurons relay these signals to sympathetic neurons in the spinal cord and trigger the activation of the postganglionic sympathetic neurons (Fig. 1). Increased sympathetic tone can regulate the activities of bone cells through their axons projected to the bone marrow and periosteum^25^. So far, the exact mechanism of how the sympathetic nerve regulates bone remodeling has not been fully revealed. The β2-adrenergic receptor is expressed by both osteoblasts and osteoclasts and is recognized as the primary receptor that mediates the action of sympathetic nerves on bone remodeling^26^. However, because the synapses between the nerve terminals and bone cells have not been clearly identified, the sympathetic modulation of bone cells may be achieved through a diffusion mechanism. Elevated sympathetic tone led to a decreased bone mass because it promoted osteoclastogenesis and inhibited the activities of osteoblasts^27^. Various nicotinic or muscarinic acetylcholine receptors of parasympathetic nerves have also been found in osteoclasts and osteoblasts. Nicotinic receptor agonist can induce osteoclast apoptosis and potentially inhibits osteoclast maturation^28^. Mice that are deficient in cholinergic receptors showed a phenotype of decreased bone mass, with the number and lifespan of osteoclasts both elevated^29^. Thus, the overall effect of parasympathetic activity on bone is likely to be anabolic.

The information of bony tissue is transmitted to the central nervous system (CNS) primarily through the dense network of sensory nerve fibers. Thin myelinated Aδ-fibers and unmyelinated C-fibers are the primary sensory nerve fibers identified in bone. Osteoblasts produce more PGE2 when bone density is declined. The elevation of PGE2 concentration in the bone initiates the regulatory mechanism of the nervous system in bone through the EP4-hypothalamus-sympathetic axis^22^. (Fig. 1) In addition to the sensory nerve-hypothalamus-sympathetic axis, sensory nerves may also directly regulate bone cells. For example, mice displayed a phenotype of decreased bone mass when the gene of semaphoring 3 A (SEMA3A) was specifically deleted in sensory neurons^30^. Semaphorin 3A has been shown to negatively affect osteoclast differentiation by forming a complex with plexin A and neuropilin1^31^.

### The roles of bone cells in sensory innervation

Among the three major types of bone cells, osteoclasts have attracted more attention for their roles in skeletal pain and sensory innervation than osteoblasts and osteocytes. Skeletal diseases with increased osteoclastic bone resorption are often accompanied by pain^32^. Osteoclasts secrete a large number of protons during bone resorption, which creates an acidic local environment. Acidosis is a prototypical noxious stimulus for nociceptive nerves that innervate bone. Acid can excite nociceptive sensory neurons through opening acid-sensing ion channels (ASICs) and the transient receptor potential channel vanilloid subfamily member 1 (TRPV1)^32^. In contrast, bisphosphonate and TRPV1 antagonist treatments alleviated bone pain-like behaviors^33^. Increased osteoclast activity and subsequent acidosis-induced bone pain have been observed in inflammatory skeletal diseases, estrogen-deficient bone loss, and osteolytic tumors^34–36^. Recent findings implicate that osteoclast also induced sensory innervation by secreting NETRIN1, a nerve attractant factor^13^. Importantly, suppressing osteoclast activity by knocking out the receptor activator of RANKL in osteocytes or treating with alendronate inhibited sensory innervations to the osteoarthritic subchondral bone, attenuated DRG neuron hyperexcitability, and reduced pain behaviors in osteoarthritis (OA) mouse models. Moreover, the researchers also showed that selective knockout of *Netrin-1* in osteoclasts exerted a similar inhibitory effect on OA pain, as compared to inhibition of osteoclast activity. Thus, osteoclast-derived NETRIN1 may play an important role in mediating OA pain.

Pre-osteoclasts can also secrete PDGF-BB to promote local angiogenesis^37^. Transgenic mice with PGDF-BB overexpression in preosteoclasts showed elevated subchondral bone angiogenesis and sensory innervation, which recapitulated certain pathologies of surgical-induced osteoarthritis^38^. Angiogenesis and sensory nerve growth are closely integrated processes. Increased axonal regeneration has been found in the vicinity of larger blood vessels^39^, and increased vascularization can facilitate axon growth in a sciatic nerve defects rat model^40^. Elevated angiogenesis is also frequently observed in arthritis pathology. In an OA mouse model with a destabilization of the medial meniscus, aberrant subchondral bone angiogenesis was developed at the early stage of osteoarthritis, and selective knockout of the gene of PDGF-BB in the preosteoclasts attenuated both angiogenesis and sensory innervation^38^. Reversely, sensory nerves also promote blood vessel growth by secreting calcitonin gene-related peptide (CGRP) and substance P^41^. These neuropeptides can regulate blood flow and stimulate endothelial cell proliferation, migration, and tube formation. Collectively, these findings implicate that skeletal diseases with high osteoclasts activity may trigger the vicious cycle between angiogenesis and nerve growth.

Unlike osteoclasts, the direct effect of osteoblasts on skeletal sensory innervation and bone pain has not been well characterized to date. It was reported recently that osteoblasts generate a nonpermissive environment for sensory innervation by secreting repelling factors and loss of neurotrophic factors expression during osteoblast differentiation^42^. Other than this, osteoblasts are more likely to affect the afferent nervous system indirectly by regulating osteoclast activities. Alterations in the expression of OPG and RANKL have been observed in skeletal diseases with high osteoblast activity, such as sclerotic bone metastases or OA^43,44^. Up-regulated sensory innervation and nociceptive neuronal excitation are then expected.

Osteocytes are terminally differentiated osteoblasts. A recent study showed that osteocytes isolated from OA patients expressed a low level of NGF. The NGF production in osteocytes was further increased when the cells were incubated in tumor necrosis factor-alpha (TNF-α)^45^. These findings implicate that osteocytes may also participate OA pain process.

## Skeleton interoception and nerve innervation

### Pain pathways

The internal state of the body was sensed by interoception, such as visceral inputs and proprioception. Whereas exteroception involves the detection of external sensory inputs such as mechanical, heat, cold, light, and sound stimuli. Anatomically, sensory pathway in vertebrates consists of peripheral nervous system (PNS) and central nervous system (CNS). Primary afferent neurons in the dorsal root ganglion (DRG) and trigeminal ganglion (TG), and neurons in nodose ganglion (NG, inferior ganglion of the vagus nerve) make up PNS. The spinal cord and brain make up CNS.

**Fig. 2:**
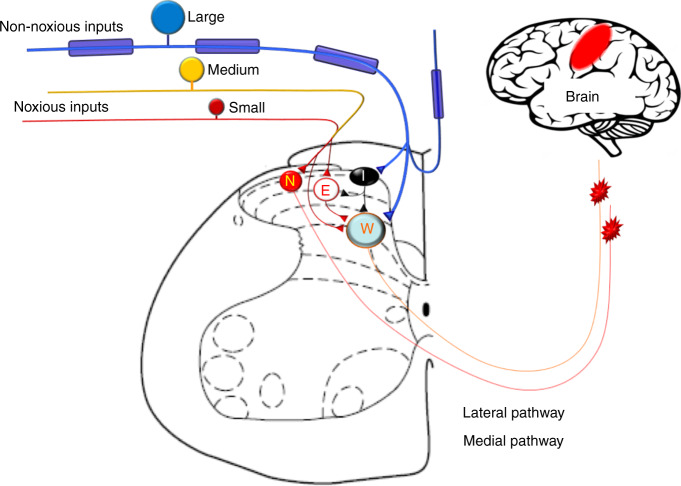
Schematic diagram illustrating the skeletal pain transmission. Bone-innervating nociceptive afferents (Aδ-, C-fibers) carry noxious inputs and mostly terminate in superficial dorsal horn, where they may activate nociceptive-specific neurons (N) and excitatory interneurons (E). Large-diameter Aβ-fibers mostly mediate non-noxious inputs and terminate in the deeper laminae and activate wide-dynamic range neurons (W) which also receive some small-diameter C-fiber inputs through polysynaptic pathways. Aβ-fibers inputs may also activate inhibitory interneurons (I) via collateral branches and induce feed-forward inhibition of other dorsal horn neurons

Primary afferent neurons are indispensable for transmitting different modalities of sensory inputs including nociceptive signals to CNS (Fig. 2). They can be separated into functionally different classes depending on modality (e.g., mechanical, thermal, chemical, or multi-modal) and responses to innocuous and noxious inputs (e.g., nociceptor)^46–48^. Innocuous mechanical stimuli such as touch, stroking, indentation, and vibration at skin mostly activate large-diameter afferent neurons (Aβ-fibers), which form synaptic contacts onto intrinsic dorsal horn neurons and post-synaptic dorsal column neurons. In DRG, many of these neurons also send collateral branches to the dorsal column nuclei. Noxious and thermal information are primarily transmitted by small-diameter (C-fiber) and some medium-diameter (Aδ-fiber) afferent neurons which can be grossly separated as peptidergic and non-peptidergic subpopulations in rodents^49–53^. For example, Mas-related G-protein-coupled receptor D (MrgprD^+^) is expressed mostly expressed in non-peptidergic neurons. Whereas calcitonin gene-related peptide (CGRP^+^) labels most peptidergic neurons^51,52,54,55^. Primary afferent neurons are pseudo-unipolar, with central processes terminating in the dorsal horn (DH) of spinal cord, and their soma are surrounded by satellite glial cells (SGCs) in the ganglion. At the spinal level, nociceptive information is processed both in the superficial lamina (I/II) and in the deep lamina (V) through poly-synaptic neural transmission^56–59^. Sensory information can be integrated and modified at the central terminals of afferent fibers, at the synaptic junctions of intrinsic DH neurons, prior to their dispatch to brain via projection neurons. The lateral pain pathway is important to the encoding of sensory and discriminatory components of pain and projects to the ventral-posterolateral nuclei of the thalamus^58–60^. Pain also has motivational and affective components, such as aversion and unpleasantness, resulting from inputs transmitted via medial pain pathway.

### Peripheral nerve innervations in bone

**Fig. 3:**
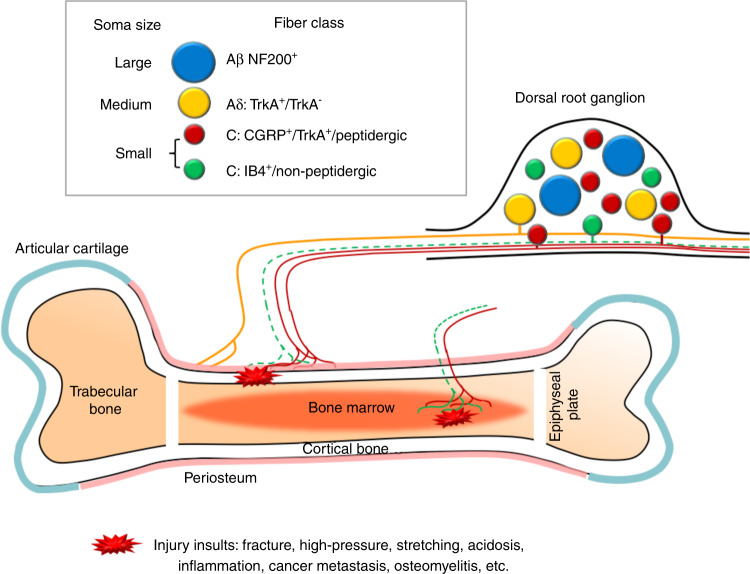
Schematic diagram illustrating sensory nerve innervation in bone. Dorsal root ganglion (DRG) contains the cell bodies of primary sensory neurons which are pseudo‐unipolar. The peripheral axons of small-diameter (red) and medium-diameter (yellow) neurons innervate periosteum and bone marrow, and can be activated by various injury insults. The majority of bone-innervating sensory fibers are unmyelinated, CGRP^+^/TrkA^+^ peptidergic C-fibers (red) from small-diameter neurons. Some thinly myelinated Aδ-fibers (yellow) from medium-diameter neurons also innervate bone. It remains uncertain whether IB4^+^/non-peptidergic C-fibers (green) also innervate bone. The centrally projecting axons of small- and medium-diameter neurons mostly terminate into superficial dorsal horn of the spinal cord. Aβ-fibers from large-diameter (blue) neurons are prevalent in the skin, but rarely found in bone. In addition to sensory fibers, bone is also innervated by sympathetic fibers (not shown) either adrenergic or cholinergic. TrkA tyrosine kinase receptor type 1; CGRP = calcitonin gene‐related peptide; IB4 = isolectin‐B4; NF200 = neurofilament 200.

Bone is well innervated by primary afferent neurons and sympathetic fibers^61,62^, which show pathological changes across several bone pain conditions, including cancer-induced bone pain (CIBP), arthritis pain, and fracture in pain animal models^1,2,63^. Bone has different compartments including bone marrow, mineralized bone and periosteum on the surface (Fig. 3). The density of nerve innervation is the highest in the periosteum, and the lowest in mineralized bone. The ratio of nerve terminal density in the periosteum, bone marrow, and mineralized bone is estimated around 1 000: 20: 1^64^.

Primary afferent neurons that innervate skin include both large-diameter Aβ-fibers, medium-diameter Aδ-fibers, and small-diameter C-fibers which can be further divided into peptidergic and nonpeptidergic subpopulations (Fig. 2). Interestingly, bone shows a different pattern of peripheral nerve innervation from that in skin. For example, bone is primarily innervated by peptidergic CGRP^+^ C-fibers and thinly myelinated Aδ-fibers^13,51,61,65–67^. The percentage of bone-innervating neurons that express tropomyosin receptor kinase A (TrkA) receptor, which mediates biological action of NGF, is much higher than those innervating other tissues including joint, skin and muscle^61,68–71^. During the development of mammalian skeletal system, NGF signaling is essential for osteochondral progenitor expansion. These findings suggest that receptors and signaling pathways on the specific subpopulation of neurons that innervate the bone may be good targets for control of bone pain. Indeed, both preclinical and clinical studies showed that antibodies against NGF have a promising therapeutic potential for OA pain and other bone pain conditions^69,71–74^. It remains to be determined whether bone is also innervated by non-peptidergic fibers, such as that identified by retrograde labeling from injection of tracer into the intramedullary space of the bone in rats^72,75^. Differences in methodology, animal species, and other experimental conditions may partially underlie the discrepancy between studies.

Bone-innervating sensory neurons also express multiple other receptors known to be important to pain transduction, such as TRPV1, ASIC3, tetrodotoxin (TTX)-resistant sodium channels (Nav.1.8), purinergic receptor (e.g., P2X3), endothelin receptor (e.g., ETAR), prostaglandin (PG) receptors, various cytokine receptors, and chemokine receptors^1,3,63,64^. Sympathetic nerve endings were found at high density in the bone marrow. Substantial evidence from both animal and clinical studies also suggests an important role of the sympathetic system and sympathetic-somatosensory coupling in bone pain^2,5,22,66,76^. Advanced behavioral test paradigms are warranted to investigate the role of the sympathetic nervous system in animal models of bone pain.

## Etiologies and manifestations of major bone pain conditions

Bone pain may be caused by common conditions such as trauma^77^, inflammation^78^, aging^2^, and by pathological conditions including OA^3,12,14,79,80^, autoimmune diseases^3,5,81–83^, genetic mutations (e.g., autosomal recessive osteogenesis imperfect)^84^, and cancer metastasis^85–88^. Some of these bone pain conditions and topics have been nicely reviewed in recent literature^1–3,5,63,69,70,72,89–93^. Like other chronic pain conditions, the manifestations of chronic bone pain include tonic background aches, sharp and breakthrough pain triggered by unknown factor, and pain associated with movement or gesture change. Compared to peripheral innervation and pain signal transduction in the skin, much less is known of those related to pain from bone and joint. So far, the origin of bone pain, and how neuronal excitability and gene expression may change are only partially known in many bone pain conditions^1–3,63,89^. Inflammation in local tissues is a common feature in several bone pain conditions which may lead to delayed wound healing, disorganized nerve regeneration, ectopic discharges and sensitization of primary afferent neurons in PNS. Gliosis, dysfunction in neuron-glia interaction, increased release of pro-inflammatory cytokine, long-lasting neuronal sensitization at spinal and supraspinal levels which underlies persistent neuropathic and inflammatory pain, also often contribute to the chronic bone pain. Many complicated bone pain conditions, such as OA pain and CIBP, have mixed etiologies which may include nociceptive, inflammatory and neuropathic components. Due to different etiologies of bone pain, mechanism-based, multi-modal therapies are urgently needed.

### Bone pain in major skeletal disorders

**Fig. 4:**
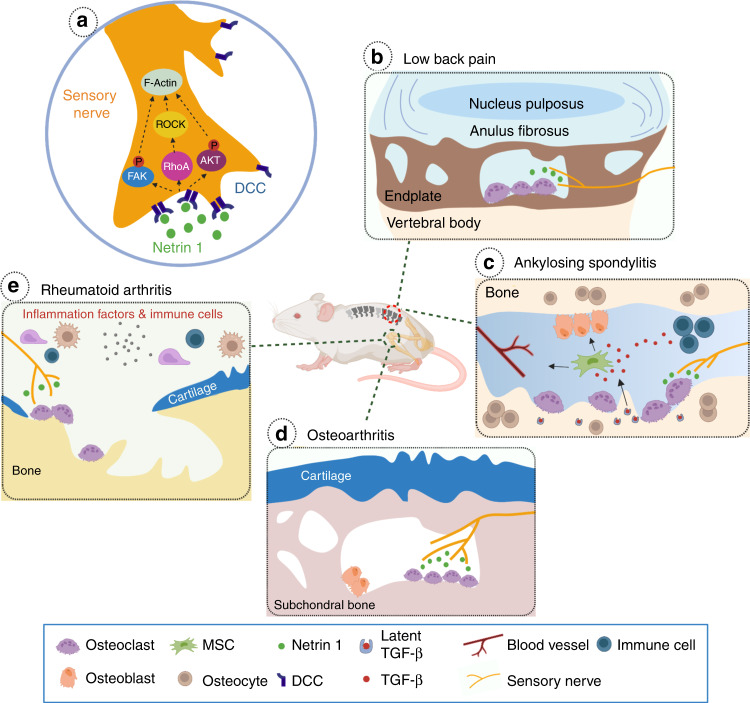
The involvement of NETRIN1/DCC signaling pathway in different skeletal diseases. **a** NETRIN1 secreted by osteoclasts bind to its receptor, deleted in colorectal cancer (DCC), to induce sensory nerve axonal growth. NETRIN1 binding to DCC triggers cytoskeletal reorganization in the nerve axon via a number of intracellular signaling cascades. **b** Sclerotic and porous endplates are common pathological changes in LBP patients. Elevated NETRIN1 secretion by osteoclasts induces sensory innervation to the porous endplates. **c** In the pathological conditions of Ankylosing Spondylitis, active TGF-β levels are elevated due to excessive secretion by immune cells and osteoclasts bone resorption. A high-level of TGF-β recruits mesenchymal stromal cells (MSCs) resulting in vessels formation and osteoblasts differentiation. In the meantime, NETRIN1 secreted by osteoclasts may also contributes to sensory innervation and bone pain. **d** Increased aberrant subchondral bone remodeling occurs during OA progression. Elevated osteoclast activity and osteoclast-derived NETRIN1 induces sensory innervation to the subchondral bone and OA pain. **e** Increased osteoclast activity and bone disruption is a typical phenotype of skeletal autoimmune diseases. Inflammatory cytokines released by the immune cells promote osteoclastogenesis and osteoclast maturation. NETRIN1 released by osteoclasts induces sensory innervation to the afflicted joint

Bone pain is a prominent feature of a range of skeletal disorders, particularly in those with increased osteoclast activity. It is also a common symptom in many genetic bone skeletal disorders featured by abnormal bone remodeling, such as Marfan syndrome (MFS), Camurati-Engelmann disease (CED), and fibrodysplasia ossificans progressiva (FOP). Secondary osteoarthritic changes in the joint have been observed in some MFS patients with prolonged protrusio acetabuli^94^. Disturbed osteoblast bone formation has been implicated to contribute to bone pain in CED, a disease that is characterized by abnormal thickening and pain in long bones^95,96^. Increased osteoblastic activity has been detected in the affected regions of CED patients. Although the exact mechanism remains unclear, quick pain relief after suppressing osteoblast activity by glucocorticoid suggests a potential role of osteoblasts in bone pain^97^. Elevated sensory innervation has been detected in the heterotopic ossification (HO) existing sites of FOP patients^98^. Activated sensory fibers from HO can initiate neuroinflammatory events by releasing neuropeptides substance P and CGRP^99,100^. This may explain the significantly higher prevalence of neuropathic pain observed in FOP patients. Moreover, the sympathetic nervous system may also contribute to bone pain resulting in complex regional pain syndrome (CRPS)^101^. In CRPS, the sympathetic deregulation disturbs the balance between vasoconstriction and vasodilation, which in turn influences the blood supply to the bone metabolism. The enhanced osteoclast activity in CRPS due to elevated sympathetic tone leads to chronic bone pain. In this section, we will discuss the recent findings regarding bone pain in skeletal diseases with high prevalence, such as rheumatoid arthritis (RA), OA, or osteolytic bone metastasis (Fig. 4).

### Bone and joint pain due to OA

Persistent background pain or breakthrough pain episodes are often associated with OA, which is a multifactorial chronic disease that often affects large joints. OA pain is also a risk factor for impaired locomotor function and mobility decline^73^. Both sympathetic fibers and sensory afferent fibers innervate bone and joint. Early electrophysiological recording showed three types of sensory afferent fibers innervating the joint, activated selectively by non-noxious movement, noxious inputs, or both^102^. Sensory neurons may develop ectopic discharges, spontaneous activity, increased excitability and responsiveness to peripheral stimulation after OA or joint inflammation^103,104^. In addition, sensory fibers which are silent under normal conditions may become activated during OA progression^105^. Nevertheless, OA joint pain involves complicated etiologies, mechanisms and origins which remain unclear. Previous studies suggested that OA joint pain is mostly mediated by thin-myelinated or unmyelinated fibers innervating tissues around the joint. These tissues including synovium^106^, ligaments^107^, subchondral bone^108–110^, and meniscus are possible origins of joint pain^109^. Synovium inflammation (synovitis) mediated sensitization of primary afferent neurons was thought to be important to OA joint pain^111^. However, synovial inflammation does not completely explain OA pain, and other sources of OA pain warrant further investigation.

Subchondral bone remodeling is increased during OA progression^112^. A recent study by Zhu et al., (2019) unraveled the role of osteoclast-initiated subchondral bone remodeling in sensory innervation for OA pain in mice^13^. Their findings showed that selectively knocking out of *RANKL* in osteocytes decreased osteoclast formation, and alendronate treatment inhibited osteoclast activity. Importantly, these treatments also inhibited aberrant subchondral bone remodeling, sensory nerve sprouting, DRG neuron hyperexcitability and behavioral pain hypersensitivity in OA mice. In addition, knockout of *Netrin1* in tartrate-resistant acid phosphatase (TRAP) positive osteoclasts also reduced OA pain behavior. In line with these findings, clinical observations showed that subchondral bone marrow edema-like lesions (BMLs) which can be observed in magnetic resonance imaging (MRI) are highly correlated with OA pain in patients^113,114^. Furthermore, inhibiting osteoclast activity by Zoledronic acid which reduced BMLs size also attenuated joint pain^115^. Collectively, these findings suggest a new cause of OA joint pain, which involves osteoclasts secretion of NETRIN1 to induce sensory nerve axonal growth in subchondral bone. Thus, the axonal guidance molecules such as NETRIN1 derived from aberrant subchondral bone remodeling may represent new targets for OA pain treatment^13^. In the subchondral bone of the distal femur, the percentage of TrkA^+^ nerve terminals was much higher than that innervating knee joint, suggesting that NGF signaling in subchondral bone may also play a role in OA pain^67,68^.

Several growth factors, in particular NGF, IGF, and vascular endothelial growth factor (VEGF)^116,117^, have been suggested to be involved in OA pain. Among these, NGF has been the most investigated and anti-NGF antibodies showed promising results in control of OA pain and other bone pain conditions^69,71,73,74^. NGF is involved in sympathetic and sensory neuron development and contributes to inflammatory pain, which is an underlying etiology in many bone pain conditions. NGF-signaling, which is mediated by TrkA and p75 receptors that are highly expressed in bone-innervating neurons, was suggested to play a more important role in bone pain than somatic pain^69,71,72^. NGF signaling may contribute to increased pain sensitivity through a variety of mechanisms. First, NGF can directly activate nociceptive sensory neurons. Second, activation of TrkA receptor by NGF can induce rapid increase in activity and function of TRPV1 and TTX-resistant, voltage-gated Na 1.8 channels through post-translational modifications, leading to increased neuronal excitability. Although TRPV1 may not contribute to the transduction of mechanical stimuli, it plays an important role in heat pain and ongoing pain^88,118–121^, and in neuronal sensitization and functional modulation of TRPA1 which is important for mechanical hypersensitivity^71,122–124^. Nav1.8 is highly expressed in bone-innervating neurons, and plays an important role in bone pain^12,57,71,125^. Third, NGF can also indirectly induce neuronal sensitization by modulating different types of immune cells, which are resident in bone marrow or are recruited to bone marrow under pathological conditions. Proinflammatory cytokines and mediators released by the immune cells may then sensitize neurons. Last, NGF can also exert effects through interactions with sympathetic neurons. Thus, NGF may play an important role in OA pain, cancer-induced bone pain, and pain after bone fracture. Yet, there is a lack of evidence for a role of NGF signaling in an inflammatory pain mode^71^. Although anti-NGF antibody exerts the beneficial effects on anti-inflammation and pain control in clinical trials, it has some side effects and safety concerns.

VEGF signaling is important to tumor angiogenesis^126,127^, and may also play an important role in bone pain conditions, including cancer-induced bone pain and OA pain. Increased VEGF level is remarkable in OA and could contribute to angiogenesis, cartilage degeneration, subchondral bone sclerosis, progression and pain associated with OA^116,117^. VEGF was found to be associated with OA progression and pain in patients with knee OA^116,128,129^. In addition, upregulation of pro-inflammatory (IL-7, IL-12, IFN-gamma) cytokines also correlated significantly with the level of pain in knee OA patients, and the synovial inflammatory mediators (IL-6, IL-8, IFN-γ, SCGF-β, CXCL1) showed significant associations to OA severity^128^. TGF-β was suggested to regulate VEGF expression in human synovial fibroblasts through both the canonical and noncanonical pathways^130^. Thus, inhibition of TGF-β and VEGF signaling (e.g., VEGFR1 and VEGFR2) may reduce OA pain and progression. Indeed, intra-articular injection of anti-VEGF monoclonal antibodies decreases joint pain in advanced OA in mice after receiving partial medial meniscectomy^116,131^. Clinical studies also showed promising results of anti-VEGF therapy for OA treatment^116,132^. VEGF also plays an important role in the delicate balance between bone formation and bone resorption which governs bone remodeling, by regulating angiogenesis and activities of osteoclastic and osteoblastic cells^129^.

PGE2 is produced from arachidonic acid by the enzymatic activity of cyclooxygenase 2 (COX-2). This inflammatory mediator can increase neuronal excitability, including enhancing the voltage-gated sodium channel Nav1.8. A most recent study by the same group of investigators further unraveled that osteoblast can secret PGE2 during aberrant subchondral bone remodeling in mice^12^. Elevated PGE2 level may then be a key contributing factor to OA joint pain and progression^22^. Indeed, genetic deletion of COX2 specifically in osteoblasts, deletion of PGE2 receptor EP4 in PNS, and genetically or pharmacologically inhibiting Nav1.8 all attenuated OA symptoms^12^. Accordingly, new therapies for OA pain may be developed by targeting aberrant subchondral bone remodeling, rebalancing PGE2 production and Nav1.8 modification.

Pain is the most prominent symptom of OA affecting over 40 million people in the US alone, but no disease-modifying anti-OA drug has been approved for treating this painful and debilitating disease. Intra-articular injections, such as hyaluronan and steroid, have commonly been used for a temporary and symptomatic relief of OA pain, but with limited efficacy. Monoclonal antibodies (mAb) to interleukine-1 (IL-1), TNF, NGF and VEGF have been under intense investigation as potential targeted therapeutics for OA, either as systemic treatments or low-dose local treatments^116,117^. If successful, these therapies may have disease-modifying effects through anti-inflammation, limiting structural progression and pain relief. Intriguingly, recent prospective clinical studies showed that human amniotic membrane and umbilical cord particulate, which were known to exert anti-inflammation and anti-scarring actions, may also exert disease-modifying actions, including pain inhibition, anti-inflammation, anti-scaring and other beneficial effects in OA joint pain. Patients with knee OA showed a significant decrease in pain score and improved physical functions following ultrasound-guided, intra-articular injection of amniotic membrane/umbilical cord particulate (CLARIX FLO®, TissueTech, Miami, FL)^133^. Accordingly, intra-articular injection of amniotic membrane/umbilical cord particulate may be a safe and effective treatment in relieving joint OA pain.

### Low back pain and bone pain during aging

The prevalence of low back pain in the US adult population is 10%–30%^134^. Aging and other pathological conditions (e.g., OA) may impair normal remodeling process and result in low back pain or spine pain, which is another major cause of morbidity worldwide, especially in elderly population. As compared to acute (<6 weeks) or subacute (6–12 weeks) low back pain, chronic low back pain (CLBP) is more bothersome and significantly reduces the patients’ quality of life. CLBP refers to persistent pain and stiffness below the costal margin and above the inferior gluteal fold, lasting for more than 3 months. CLBP is the second leading cause of disability, and its prevalence continues to increase in recent decades. The exact etiology of CLBP is not yet determined and many anatomic structures (e.g., nerve roots, soft tissue, vertebrae, zygapophyseal and sacroiliac joints, and intervertebral discs) may serve as the potential origin of pain. Psychological factors, such as stress, depression, and/or anxiety, also predispose to or interact with the pain sensation in the low back region. CLBP is mostly generated by muscle tension and spasm. Other pathological alterations such as facet joint arthritis, disc degeneration, spinal stenosis, or radicular compression account for the rest portion of CLBP cases.

Although many low back pain conditions do not have clear pathoanatomical origins and underlying mechanisms, degeneration of intervertebral disc and facet joints were thought to be one of the main causes. Facet pain usually derives from arthritic alterations of the joints or the stress within the joint capsule^135^, representing as unilateral or bilateral low back pain that can be exacerbated by lumbar extension, prolonged standing or sitting, changes in physical activities, and psychosocial stressors^136,137^. The prevalence of facet joint OA also increases with age and the L4-L5 facet joints are the most afflicted joints. Women show a higher prevalence of lumbar facet joint OA relative to men^138^. Moreover, the presence of disc degeneration and facet pathology in older adults is very common. A case-control study showed that higher radiographic severity scores were associated with the presence of CLBP but not associated with the pain severity^139^.

Radicular pain is another common type of CLBP evoked by ectopic discharges of DRG neurons, referring to pain that radiates from the back and hip into the leg accompanied by numbness, tingling, and muscle weakness. Disc herniation is the leading cause of dorsal root compression and subsequent abnormal nociceptive neuron firing in the DRG. Other causes include narrowing of the spinal canal, neural foramen, lateral recess dorsal root compression, or inflammation^140–142^. Both chronic disc degeneration and acute injury to the spine can induce disc herniation. The main function of the intervertebral disc is to absorb mechanical shock, preserve spinal movements and distribute torsional and axial forces. It has been demonstrated by a recent publication that physiological mechanical stress promotes the transport of parathyroid hormone 1 receptor (PTH1R) to the cilia and enhances parathyroid hormone (PTH) signaling in nucleus pulposus cells^143^. PTH can promote TGF-β activation by inducing the transcription of integrin α_v_β_6._ The TGF-β-connective tissue growth factor (CCN2)-matrix protein signaling cascade is then activated for the maintenance of intervertebral disc homeostasis. However, abnormal mechanical stress, overuse, and aging may induce degenerative alterations in the nucleus pulposus or surrounding annulus fibrosus and subsequent disc herniation. The healing of the annulus fibrosus and nucleus pulposus is usually accompanied by neovascularization and penetration of the minute sensory nerves, leading to mechanical and chemical sensitization^144^. It has been reported that aberrant mechanical loading leads to accelerated ossification and hypertrophy of vertebral end plate and inter-vertebrate disc degeneration^145^. Therefore, pathological alterations in the vertebral end plate are also essential treatment targets for CLBP. Indeed, porosis and elevated sensory innervation within the endplates were observed in both lumbar spine instability and aging mouse models^125^. A recent study by Ni et al. suggested that osteoclasts-induced sensory innervation in the porous areas of sclerotic endplates may contribute to spine pain^125^. Osteoblast-secreted PGE2 was shown to activate bone-innervating sensory neurons and tune down sympathetic tone for osteoblastic bone formation^22^. However, elevated PGE2 levels from increased osteoblast activity may lead to sodium influx through Nav1.8 channels, and thus activate nerve terminals and induce pain. This mechanism was also shown to contribute to OA joint pain^12^. Osteoclast resorption likely causes the porosity of sclerotic endplates. During aging, the porosity of the sclerotic endplates may resemble low bone mineral density, which stimulates osteoblast secretion of PGE2 and promote aberrant sensory innervations causing pain. Intriguingly, inhibition of osteoclast formation by knocking out *Rankl* in the osteocytes attenuated aforementioned pathological changes and reduced pain, suggesting potential new therapeutic targets for low back pain^125^.

Current treatments of low back pain are all palliative without treating the causes of underlying degeneration and etiology, and effects are short-lived. Similar to the inhibition of OA pain following intra-articular drug injection, intradiscal injection of amniotic membrane and umbilical cord particulate also induced satisfying pain relief without adverse effects in patients with discogenic low back pain^146^. Furthermore, intra-articular injection of particulate also reduced back pain caused by facet joint syndrome^147^. Thus, future strategies to inhibit local nerve abnormal innervation, limit disc degeneration, and promote regeneration with stem cell therapy, progenitor cell and tissue transplantation may improve the treatment of this common health problem.

### Pain in bone autoimmune diseases

Pain is the most important symptom of RA, which often persists even with optimal control of inflammation, indicating RA pain may arise from mechanisms other than inflammation. RA patients with active inflammation often describe their pain symptoms as ‘aching’, ‘sharp’, ‘tender’ or ‘tiring’ and ‘sickening’ according to the McGill pain Questionnaire^148^, The levels of inflammatory cytokines such as IL-1β, IL-6, TNF-α or growth factors such as vascular endothelial growth factor, nerve growth factor, or chemokine CCL2 are elevated in the RA synovium or synovial fluid. These factors excite or sensitize nociceptive nerves, by evoking excitatory inward currents, modifying the function of ion channels, or promoting nerve growth^149^. Recent studies showed that somatostatin and anti-inflammatory cytokines IL-4 and IL-10 potentially reduced neuronal sensitivity^150–153^. These factors were also detected in the synovium system of RA patients^154^. Although their ability to moderate RA pain remains to be determined, it sheds new light on exploring the therapeutic strategy of RA pain. Moreover, elevated osteoclast activity is one of the hallmarks of RA. This pathological change is somewhat similar to that observed in subchondral bone in early phase of OA and in endplate during spinal degeneration^13,125,155^. Thus, the osteoclast-NETRIN1 axis or PGE2-EP4- Nav1.8 axis is likely involved in the mechanism of subchondral bone-derived RA pain, but this hypothesis has yet to be systematically tested in RA.

RA animal models indicate that altered neuronal plasticity may also contribute to RA pain. Indeed, many RA patients experience ‘burning’ on light pressure, a classic symptom of neuropathic pain. Neuropathic-like symptoms in RA might be attributable to abnormal central pain processing, which generates a widespread reduction in pain threshold in response to pressure, not only over the inflamed joints but also at non-articular sites^156,157^. Central sensitization may occur at spinal or supraspinal levels in RA patients. Increased electroencephalographic activity has been reported in response to repeated stimuli in RA patients^158^. Interestingly, functional MRI scan showed that applying mechanical forces to the RA joints can activate brain regions in the thalamus, secondary sensory cortex, and some regions in the limbic system^159^. These regions are involved in either pain processing or emotional processing. This finding may explain why the psychological state modulates the evoking of RA pain. For example, activity in the medial prefrontal cortex in response to joint palpation was augmented in people with depression. The low mood has been reported to associate with augmented pain processing through increased activity in the limbic system and descending facilitation of nociceptive transmission.

Ankylosing spondylitis (AS) is another common type of chronic autoimmune disease that affects the skeletal system. Chronic back pain is the major symptom of AS in addition to progressive spinal rigidity and inflammation of the hips, shoulders, and peripheral joints. HLA-B27 has been shown to be associated with AS, although the connection between them remains to be elucidated. A previous study shows that HLA-B27-specific CD8^+^ T cells can be activated by antigenic components of infectious bacterial pathogens that partially resemble HLA molecules^160^. Activation of CD8^+^ T cells further leads to autoreactivity and autoimmune response. Similar to that of RA, high levels of inflammatory cytokines released by immune cells lead to the excitation and sensitization of nociceptive nerves and AS pain. A recent finding shows that the inflammatory microenvironment in AS facilitates chondrogenesis and cartilage calcification in spine ligaments. Inflammation also promotes osteoclast differentiation and maturation. A large amount of active TGF-β is released during osteoclastic resorption of the calcified cartilage and bony tissue, which triggers the ossification in a similar pathological process of acquired heterotopic ossification^161^. Similar to the pathological conditions of OA, elevated osteoclast activity may contribute to sensory nerve growth, nociceptive excitation, and the development of back pain in AS patients.

### Bone pain from fracture

Trauma such as fracture, stressful impact and mechanical distortion often causes excruciating bone pain, mediated by nerve fibers innervating the injured bone. The healing process is accompanied by dull aching pain, and movement or pressure may cause sharp pain. DRG neurons that innervate bone structures are primarily thin-myelinated Aδ-fibers and unmyelinated C-fibers which are mostly peptidergic^61,62^. These neurons are mechanosensitive but demonstrate higher activation thresholds than skin innervating mechanosensitive neurons (e.g., Aβ-fiber)^102,162^. DRG neurons generate injury discharge immediately after injury and may last for hours, which could sensitize neurons in CNS.

Pro-inflammatory cytokines may exacerbate pain responses after bone fracture^163,164^. Chemokines and pro-inflammatory cytokines (TNF-α, IL-6, and IL1β) may be released from fracture site as part of innate immune responses to injury, which then attract circulating immune cells and inflammatory cells leading to local inflammation. These detrimental changes in bone microenviornment may increase osteoclast activity and bone resorption, increase PGE2 level, delay natural healing processes, sensitize afferent sensory neurons and induce pain hypersensitivity^12,14,22,125,165,166^. Accordingly, nonsteroidal anti-inflammatory drugs (NSAIDs) and acetaminophen have been the common analgesics for pain control at the acute stage of traumatic fracture by attenuating the inflammatory responses^167^.

Ectopic sprouting of the sensory and sympathetic nerve fibers at the fracture site may also exacerbate and prolong the pain after fracture. Although sequestering NGF may alleviate bone pain conditions including pain-like behaviors in animal models of skeletal fracture, anti-NGF treatment is not recommended for stress fracture pain due to delay of nerve regrowth and fracture healing^168–170^. Both NGF and VEGF signaling had been shown to play an important role in bone repairing after fracture^168–170^. NGF expression was increased in periosteal stromal progenitors and fracture-associated macrophages, as well as increased sprouting of TrkA^+^ nerve fibers^169^. Inhibition of TrkA function decreased sensory innervation, revascularization, and ossification of the fracture callus, which are important to bone repair. Pain often gradually dissipates after normal healing process of the fracture is completed. However, pain may become chronic in some patients, which involves central sensitization and other maladaptive changes.

### Pain from bone metastasis

Pain is often associated with advanced cancer and affects 60%–80% of these patients^171,172^. CIBP is often caused by bone metastasis of other carcinomas especially tumors originating in the breast and the prostate, and also resulted from primary bone and bone marrow neoplasm such as multiple myeloma. The prevalence is high in patients after bone metastasis, especially those with terminal breast, prostate, lung and kidney cancers^88,89,173^. Multiple myeloma, the most common primary bone tumor, commonly causes generalized lytic bone lesions and more than 2/3 of these patients experience debilitating bone pain. The high prevalence of metastatic CIBP in bone metastasis and bone/bone marrow centered hematolymphoid malignancies significantly reduces the quality of life, impairs functions and increases risks of pathological fractures^1,4,86^. So far, our knowledge about CIBP remains incomplete, and effective clinical treatment is lacking. Findings from animal models have contributed a large amount of knowledge to the mechanisms of CIBP, and progress in both preclinical and clinical studies of CIBP have been extensively reviewed in recent years^1,2,5,87–89,92,173–178^.

Manifestations of CIBP are complex and diverse. Though in multiple myeloma, CIBP is usually associated with disease progression, relapse and refractory, pain severity may be variable in different diseases and different stages. In cancer metastasis, vertebrae (69%) and the pelvic bones (41%) are more affected than the long bones (25%) and skull (14%)^179^. However, there are no clear correlations between the pain severity/chronicity with the location, size and severity of metastatic bone damages, or with tumor type and size^88,89,173,178^. The reasons for this large diversity and individual differences remain unclear. Initially, CIBP pain may be felt as mild, short-lasting background pain. The severity, frequency and chronicity of pain gradually increase during cancer progression and change from intermittent dull aches to continuous, ongoing pain. The intensity increases at night and is exacerbated by movement, and patients may also develop somatic tactile hypersensitivity^86,88,89,173,178^. In the later stage, cancer pain may be a result of cancer progression due to expansive growth of rapidly proliferative tumor cells in confined marrow space, compression and invasion of nerve, plexuses or other neurological tissues, and involves recurrent episodes of breakthrough pain which is spontaneous piercing, and unbearable^88,180^. CIBP may also be iatrogenic resulting from anti-cancer treatment (e.g., chemotherapy and radiotherapy-induced peripheral neuropathy).

Extensive efforts have been made in understanding mechanisms of CIBP, with progress achieved through extensive research in animal models in past decades^85,174,181,182^. Numerous animal models have been developed to mimic the clinical characteristics of various CIBP pain by injections of various types of cancer cells (e.g., MatLyLu prostate cancer, Lewis Lung cancer, melanoma) into bone marrow of the tibia or femur bone in rodents^85,87,88,183^. Hypersensitivity to mechanical stimulation at the limb is common in these models. Comparatively, levels of thermal hypersensitivity, movement-related pain and spontaneous pain are more variable among different studies.

Metastatic CIBP has complex pathophysiology and includes nociceptive, inflammatory and neuropathic components^88,89,173^. It shares some common cellular and molecular mechanisms with chronic inflammatory and neuropathic pain conditions, including a state of neuronal hyper-excitability at spinal and supraspinal levels (central sensitization) and in primary sensory neurons (peripheral sensitization), increased activation of glial cells including both astrocytes and microglial cells in the spinal cord, heightened neuron-immune responses, increased levels of pro-inflammation cytokines such as IL-1b and TNF-α and dysfunction in neuron-glia interactions. In line with this notion, NSAIDs, anti-inflammatory drugs, and anti-NGF antibodies may partially alleviate CIBP in animal models^1,5,88,184^. Nevertheless, metastatic CIBP has important mechanisms that differ from other bone pain conditions. In particular, pathological changes in bone marrow microenviornment is pivotal to the development and persistence of CIBP. These changes include complicated interactions between tumor cells, osteoclast and osteoblast cells, immune cells and neuronal innervation at the site of bone metastases.

After escaping from the primary tumor site, invading and colonizing the bone, tumor cells may release endothelin (ET) and other mediators which increase osteoblast activity. Activated osteoblasts release RANKL, which in turn triggers the proliferation and formation of osteoclasts, causing bone destruction through osteoclast-mediated osteolysis^13,185^. Osteoclasts and tumor cells also induce local acidosis by releasing ATP and acidosis-causing H^+^, which can stimulate nerve terminals and cause pain by activating purinergic P2X receptors and opening ASICS3 channels. Inflammatory cells including macrophages and mast cells are also recruited and joined the microenvironment. They increase the release of pro-inflammatory cytokines and mediators (e.g., endothelin, bradykinin, proteases, IL6, NGF, prostaglandins, TNF-α), many of which are known powerful activator and sensitizer to neurons^88,175^. During bone metastases, growth factors including NGF and brain-derived neurotrophic factor (BDNF) are released from stromal cells, immune cells (e.g., mast cells, macrophages) and tumor cells. These growth factors can activate macrophages to release pro-inflammatory cytokines. Importantly, they also act alone with other mediators and VEGF which are important to tumor angiogenesis, induce aberrant nerve innervation in periosteum, mineralized bone tissue, and the bone marrow, which is important for increased neuronal excitability and CIBP. The pathological growth of nerve fibers is extensive and may exceed the normal density of nerve innervation by > 10 times^185,186^

Currently, CIBP is managed by using the three-step ladder World Health Organization (WHO) model, which is devised in 1982. This is not only dated but also leaves over 20% of cancer patients with unsatisfactory pain control. Nonopioid analgesia, such as NSAIDs, one of the first ladder choices is contraindicated in multiple myeloma patients because of the high risk of renal failure^187^. The opioids remain the first-line. However, side effects are prominent^6–8^. Due to multifactorial etiology of CIBP, both disease-modifying anticancer therapies and symptomatic drug treatments are needed to improve quality of life. Yet, the underlying etiology of CIBP remains very challenging to correct, and hence developing safe, non-opioid symptomatic treatment is particularly an urgent need^9,86,181^. Due to large variations in cancer progression, pain states and intensities in patients, management of CIBP also may need multi-modality, multi-disciplinary, and individualized therapies^183,188^.

## Prospects of bone pain treatment

### Peripheral targets for bone pain treatment

Our review describes recent advances in pre-clinical studies of peripheral mechanisms of bone pain in different pathological conditions, which are pervasive problems that cause substantial suffering and burden. Available drugs for treatment of bone pain conditions mostly target receptors expressed broadly in the body, and are often associated with CNS side effects. In particular, opioid analgesics can cause severe central adverse effects such as sedation, addiction and abuse, especially with prolonged usage. Accordingly, developing safe, non-opioid therapies for bone and joint pain is urgent and a high research priority. Increasing amounts of evidence support the tenet that increased excitability in primary afferent neurons, as well as close interactions between the neuron and different types of non-neuronal cells (immune cells, osteoclast, osteoblast) in the bone microenvironment play critical roles in various bone pain conditions.

Mounting evidence suggests that peripheral neuronal and non-neuronal mechanisms contribute to clinical pain conditions, including bone pain^6,69,70,72,120,165,189^. Because the peripheral “pain generators” in bone pain states of different etiologies may represent “low-hanging targets” for drug development, identifying new targets in the periphery nervous system and local tissues which are important to the development and maintenance of bone pain merits particular attention^189^. Pharmaceutical or non-pharmaceutical interventions that can reverse or halt peripheral pathological alterations also hold the promise to mitigate skeletal disease-related pain. For example, approaches targeting matrix-degrading proteases or senescent cells, promoting cartilage repair, limiting bone modeling, or suppressing local inflammation have been shown to alleviate OA pain in some preclinical studies^190,191^. However, effective disease modifying drugs are still not available, and NSAIDs remain the first-line therapy for most skeletal degenerative diseases induced bone pain, such as OA, disc degeneration, and many genetic bone diseases. Although NGF inhibitors have been introduced to many skeletal diseases for pain control^192^, they also increased the risk of rapid progress of OA in some patients treated with high doses^193^. In RA and AS, TNF inhibitors, glucocorticoids, and methotrexate were shown to reduce pain symptoms^194–196^. However, cure is still rare for autoimmune-induced skeletal diseases, even with the effector cytokines being neutralized. Individuals with RA frequently suffer from relapse, particularly when the treatment is interrupted or stopped^197^. Crosstalk between the immune system and nociceptive pathways is central to inflammatory pain conditions^141^. Therefore, new therapies might target the interplay between activations of immune system and nociceptive neurons.

For neuropathic pain control, compared to drugs (e.g., opioids, gabapentin) that primarily rely on attenuating central mechanisms of pain, targeting peripheral mechanisms for pain control has several benefits and advantages^189^. For example, suppressing peripheral “pain generators” will effectively inhibit “pain at its source”, and also avoid potential side effects due to central drug actions, and thus a safer treatment. Peripherally acting mu-opioids were shown to attenuate both evoked pain and ongoing pain-related behavior in animal models of neuropathic pain^6,120,121,198,199^. However, the detrimental effects of mu-opioids, such as morphine, on bone, cancer progression and immune responses may limit its use in bone pain conditions^5,200^. Intriguingly, a kappa-opioid, U50448, was shown to attenuate CIBP including tumor-induced spontaneous flinching in animal models, without altering bone loss and tumor burden^181^. Peripherally acting kappa-opioids are being developed and have demonstrated inhibitory effects on pain, neurogenic inflammation and nociceptor sensitization in animal studies^201^. Another promising candidate is the peripherally restricted agonist to type 1 cannabinoid receptors (CB1), which has the potential of attenuating hyperexcitability of primary afferent neurons and several chronic pain conditions, including CIBP in animal models^202–208^.

### Challenges in the study of bone pain with using animal models

A variety of animal models have been developed to simulate pathological process and clinical manifestations of different bone pain conditions, and are widely studied for the underlying biological mechanisms^12–14,77,87,88,118,209,210^. Taking CIBP for an example, nearly 40 different models have been described by using injections of different types of cancer cells (e.g., NCTC 2472 fibrosarcoma, Walker 256 carcinoma, MRMT-1 mammary gland carcinoma) into the tibia or femur bone^85,87,88,183^. Findings in animal models have contributed important knowledge to the mechanisms of bone pain. Nevertheless, translation of preclinical findings to development of novel pharmacological therapies for clinical use remains fraught with multiple challenges^211,212^.

Bone and joint pain in animal models are usually inferred by measuring paw withdrawal responses to external test stimulation. In particular, mechanical stimulus-evoked behavioral responses have been the most commonly measured outcome. Yet, withdrawal responses represent a spinal reflex to test stimulation, and altered withdrawal reflex may reflect change in muscle and motor functions, especially in bone and joint pain conditions^69,87,88^. Thus, changes in mechanical reflex behavior alone may not truthfully indicate bone pain perception in patients. In addition to mechanical hypersensitivity, patients often experience ongoing pain, background pain, spontaneous pain and movement-induced pain associated with pathological changes in bone and joints, and are the most bothersome and difficult to control. These important clinical symptoms have been difficult to measure in animal studies^88,212–214^. This partially contributes to a lack of extensive pre-clinical investigations focusing on these bone pain manifestations, which may have different mechanisms from that underlying the evoked pain hypersensitivity.

Evidence from studying other pain conditions implies that behavioral tests of reflex responses alone do not correlate well with effectiveness of drug treatment in patients^189,211,212^. Although the reasons for this lack of translatability may be multi-factorial, insufficient predictive ability to measure reflex responses alone in animal models may be an important one. Accordingly, comprehensive assays using non-reflex outcome measures, such as cerebral-dependent conditioned place preference, place escape or avoidance paradigm, operate behavior test, voluntary wheel running activity and gait analysis are warranted for evaluating effectiveness and studying the mechanisms of new therapies for alleviating ongoing pain and movement-related manifestations of bone and joint pain, in order to facilitate future translational studies. This may help bridge gap between new findings in preclinical mechanistic study and the development of new clinical treatments^211–213^.

## Conclusions

The ongoing knowledge gap and challenges remain substantial in a complete mechanistic understanding of various bone pain conditions, and especially in translating findings from animal models to clinical treatment. Bone pain in clinic may have various aspects and often involve mixed etiologies. Examination of specific subpopulations of DRG neurons innervating bone and joints in human is warranted. Single cell RNA-sequencing studies showed that DRG neurons express different transcription profiles, and show different changes after axonal injury^49,215^. Yet, details remain unclear on how bone-innervating sensory neurons and sympathetic ganglion neurons differ in their transcription profiles from those innervating skin. In addition, the neurochemical and electrophysiological properties of bone-innervating DRG neurons, especially those responding to mechanical load, have not been well characterized. New mechanism-based therapies may fundamentally improve bone pain treatment. Although some of these knowledge gaps may be challenging to fill, the clinical need to improve bone pain treatment demands continuing research efforts to develop new therapies with multi-mode of action to target the underlying mechanisms of different bone pain conditions.
